# Genetic Profile and Clinical Characteristics of Brugada Syndrome in the Chinese Population

**DOI:** 10.3390/jcdd9110369

**Published:** 2022-10-28

**Authors:** Lin-Lin Wang, Yang-Hui Chen, Yang Sun, Man Huang, Hao-Ran Wei, Hao Liu, Ke Xu, Xiu-Li Song, Peng Chen, Lun Tan, Jin Huang, Zong-Zhe Li, Rui Li, Ting Yu, Fei Ma, Hu Ding, Yan Wang, Dao-Wen Wang, Hong Wang, Chun-Xia Zhao

**Affiliations:** 1 Division of Cardiology, Departments of Internal Medicine, Tongji Hospital, Tongji Medical College, Huazhong University of Science and Technology, Wuhan 430030, China; 2 Hubei Key Laboratory of Genetics and Molecular Mechanisms of Cardiological Disorders, Huazhong University of Science and Technology, Wuhan 430030, China; 3 Genetic Diagnosis Center, Tongji Hospital, Tongji Medical College, Huazhong University of Science and Technology, Wuhan 430030, China

**Keywords:** Brugada syndrome, genetic testing, *SCN5A*, electrocardiogram, arrhythmia

## Abstract

Background: Brugada syndrome (BrS) is an inheritable arrhythmia syndrome that can lead to sudden cardiac death in patients while the heart structure is normal. However, the genetic background of more than 65% of BrS probands remains unclear. Objectives: The purpose of this study is to report the variant spectrum in a Chinese cohort with suspected BrS and to analyze their distinct clinical and electrocardiographic features. Methods: Patients with suspected BrS from Tongji Hospital between 2008 and 2021 were analyzed retrospectively. Results: A total of 79 probands were included in this study. Patients with type 1 BrS electrocardiogram (ECG) had a prolonged QRS duration compared to patients with type 2/3 BrS ECG. Of them, 59 probands underwent genetic testing. Twenty-five patients (42.37%) showed abnormal genetic testing results, and eight of them (13.56%) carried pathogenic/likely pathogenic (P/LP) mutations. Mutation carriers presented much more prominent depolarization and repolarization abnormalities than non-carriers, including a prolonged P-wave duration, QRS duration, QTc interval, decreased QRS amplitude, and deviation of the electrocardiographic axes (T-wave axis and R-wave axis). Furthermore, our study identified four novel P/LP mutations: Q3508X in *TTN*, A990G in *KCNH2*, G1220E, and D372H (in a representative pedigree) in *SCN5A*. Conclusions: Our study showed the variant spectrum of a suspected Chinese BrS cohort, and we identified four novel P/LP mutations in *TTN*, *KCNH2*, and *SCN5A*.

## 1. Introduction

Brugada syndrome (BrS) is one of the most common inherited primary arrhythmia syndromes with an extensive genetic heterogeneity. BrS is definitively diagnosed when a type 1 ST-segment elevation is observed either spontaneously or after an intravenous administration of a sodium channel blocking agent in at least one right precordial lead (V1 and V2), which are placed in the 2nd, 3rd, or 4th intercostal space [1]. The prevalence of BrS varies among continents, countries, and ethnicities, and is highest in Southeast Asia (0.37%) [2], which may be attributed to genetic polymorphisms in the *SCN5A* promoter region [3]. However, the real prevalence in the general population remains unclear due to the intermittent and concealed classic electrocardiogram (ECG) pattern [4]. 

BrS typically manifests in the third or fourth decade of life, but this syndrome may occur at any age, from 2 days old to 84 years old. The incidence in males is 8–10 times higher than that in females [4]. More than 60% of patients with a BrS ECG are asymptomatic and diagnosed incidentally by a routine evaluation, family screening, or the observation of an abnormal ECG pattern during a fever [5]. A small number of patients present various symptoms, including slight darkness, a history of syncope (30%), paroxysmal nocturnal dyspnea, ventricular tachycardia/fibrillation (VT/VF), and sudden cardiac death (SCD) (6%). An SCD is often the first manifestation of BrS, predominantly in adult males at night or during rest [6,7]. BrS is considered to be responsible for at least 4% of all SCDs and at least 20% of those occur in patients with normal hearts [4]. Patients with aborted cardiac arrest or documented spontaneous sustained VT are at the highest risk of an SCD, followed by a history of cardiac syncope. Moreover, the risk of life-threatening arrhythmias in asymptomatic patients is 0.5–1.5% per year [6]. From this, it is important to focus on identifying the genetic cause of BrS to detect asymptomatic genetic carriers at risk of an SCD.

BrS was initially thought to be a monogenic, autosomal dominant disease. However, the occurrence and prognosis of BrS are more likely affected by a combination of multiple genetic alterations and environmental factors due to the incomplete penetrance and variable expressivity [7]. Currently, more than 500 potentially disease-causing variants account for about 30–35% of BrS patients, mainly including genes regulating the sodium current (INa), the L-type calcium channels (ICa), and the transient outward potassium channels (Ito) [8]. The majority of all pathogenic mutations (>75%) reported are located in *SCN5A*, which is the only definitive gene for BrS, accounting for 20–30% of BrS patients [9,10,11]. About 150 additional variants proposed to be causative of BrS in other genes explain no more than 10% of cases. These genes are classified as minor genes with a limited evidence for BrS [7,11]. Thus, approximately 65–70% of BrS probands remain genetically undetermined [7].

Genetic testing is recommended for an early detection of a patient’s relatives who are potentially at risk [4]. However, the data about the genetic background and clinical characteristics of Chinese BrS patients are scarce [12,13,14]. It has been reported that the prevalence rate of *SCN5A* mutations is around 8% (4/47) in Taiwan [14] and 14% (5/36)–34% (22/65) in Hong Kong [12,13]. Additionally, the distribution of disease-causing genes among BrS patients in the Asian population might differ from that in the Caucasian population (20–25%) [15]. However, there is a lack of large-scale genetic and clinical characteristic data in Chinese BrS patients. In the present study, we aimed to determine the prevalence and spectrum of genetic variations in BrS-susceptibility genes in a Chinese cohort with suspected BrS, and to analyze the clinical and genetic features.

## 2. Methods

### 2.1. Study Population

A total of 79 Chinese probands with suspected BrS were included from Tongji Hospital Affiliated Tongji Medical College of Huazhong University of Science and Technology (Wuhan) between 2008 and 2021. 

Patients were suspected to have BrS according to the following diagnostic criteria [5]: patients showed one of the three types of BrS ECG and/or presented with one of the following typical symptoms: documented VF or polymorphic VT, arrhythmic syncope or paroxysmal nocturnal dyspnea, a positive cardiac electrophysiology examination, a family history of SCD at <45 years old or type 1 BrS ECG in family members. 

Type 1 (“coved type”), the only diagnostic pattern for BrS, is defined as an ascending and high take-off of ≥2 mm at the end of the QRS duration in ≥1 right precordial leads (V1 to V3), followed by a coved or rectilinear down-sloping ST-segment and a negative symmetric T-wave. Type 2 (“saddle-back type”), a suggestive pattern of BrS [16], is characterized by an ST-segment elevation ≥0.5 mm (generally ≥2 mm in V2) in a ≥1 right precordial lead (V1 to V3), followed by a convex ST and a positive T wave in V2 or variable morphology in V1. Type 3 is characterized by either a saddleback or coved appearance with an ST-segment elevation <1 mm. Similarly, a type 3 ECG is only suspected of BrS. Besides these, we collected other atypical clinical manifestations of patients as supportive diagnoses, including a first-degree atrioventricular block, atrial fibrillation, positive late ventricular potentials, and fragmented QRS. Furthermore, other causes of the ST-segment elevation were excluded, such as an atypical right bundle branch block, ventricular hypertrophy, early repolarization, acute pericarditis/myocarditis, acute myocardial ischemia or infarction, arrhythmogenic right ventricular dysplasia, hypothermia, dissecting aortic aneurysm, pulmonary thromboembolism, and Duchenne muscular dystrophy and so on [17].

We finally recorded the following clinical features: (1) sex; (2) the age at diagnosis; (3) the type of BrS ECG; (4) the symptoms, including typical and atypical symptoms [1]; (5) a documented VT/VF; (6) the syncope; (7) any family history of SCD; and (8) the implantation of an implantable cardioverter defibrillator (ICD).

### 2.2. ECG Measurements

The measurements of the ECG parameters were automatically read by machines and calibrated manually, including (1) the heart rate; (2) P-wave, QRS, and T-wave duration; (3) PR, QT, and QTc interval; (4) P-wave, QRS, and T-wave axis; (5) R-wave and S-wave amplitude in lead V1 and V5; (6) RV5 + SV1; and (7) RV1 + SV5.

### 2.3. Genetic Testing

Of the 79 probands, 59 underwent genetic testing. Twelve patients performed whole-exome sequencing (WES) and the other 47 were analyzed with target sequencing using a gene panel associated with arrhythmias and cardiomyopathies. Genomic DNA was extracted from the peripheral blood lymphocytes of 59 probands using the DNA blood mini kit (TIANGEN, China). All gDNA samples were of a high quality with a DNA concentration >20 ng/μL and an OD 260/280 from 1.8 to 2.0. The WES was conducted on an Illumina HiSeq X and NovaSeq System, and then the obtained data were processed according to the Genome Analysis Toolkit Best Practices recommendations, as we previously described [18]. Target sequencing was performed using the Ion Torrent platform (Thermo Fisher, Carlsbad, CA), as previous reported [19]. Finally, 29 genes associated with BrS were analyzed, including *ABCC9*, *AKAP9*, *ANK2*, *CACNA1C*, *CACNA2D1*, *CACNB2*, *CASQ2*, *DSG2*, *DSP*, *GPD1L*, *HCN4*, *KCND3*, *KCNE3*, *KCNE5*, *KCNJ8*, *KCNH2*, *PLN*, *PKP2*, *RANGRF*, *RYR2*, *SCN10A*, *SCN1B*, *SCN2B*, *SCN3B*, *SCN4A*, *SCN5A*, *SCNN1A*, *TRPM4*, and *TTN*.

### 2.4. Variants Screening

The variants were annotated using ANNOVAR [20] and classified according to the American College of Medical Genetics and Genomics (ACMG) guidelines [21]. We screened variants in two parts. First, we searched all the reported variants in the ClinVar database and PubMed, and excluded benign or likely benign variants. Second, we screened the novel variants according to the following criteria: (1) functional variants including exonic, splicing, nonsynonymous SNV, insertion, deletion, substitution, and stopgain; (2) having a minor allele frequency (MAF) ≤ 0.1% in the ExAC and GnomAD; (3) more than 8 of 14 in silico tools predicting “damaging” or “probably damaging”, including SIFT, Polyphen2, LRT, MutationTaster, MutationAssessor, FATHMM, PROVEAN, MetaSVM, MetaLR, M-CAP, REVEL (score > 0.5), CADD (score > 20), fathmm-MKL, and GERP (score > 4); and (4) excluding verified false-positive variants by Sanger sequencing.

### 2.5. Sanger Sequencing

Sanger sequencing was used to confirm all the rare variants screened by the above methods. The PCR primers were designed by Primer Premiers 5.0 and listed in Table S1.

### 2.6. Waterfall Plot and Needle Plot

A summary waterfall plot of the variants in the genes associated with BrS was generated using the R package “maftools” (R Foundation for Statistical Computing, Vienna, Austria). A mutation needle plot of *SCN5A* was created with MutationMapper (https://www.cbioportal.org/mutation_mapper, accessed on 6 July 2022).

### 2.7. Protein 3D Structure Prediction 

The structural change in the protein by the substitution of the amino acid was predicted using Missense3D (http://missense3d.bc.ic.ac.uk/missense3d/, accessed on 27 August 2022). This tool uses three-dimensional structural information from experimentally determined protein models to predict the consequences of amino acid substitutions. The variant was analyzed using the experimentally determined structure of the sodium channel protein type 5 subunit alpha (UniProt ID: Q14524, PDB code: 6LQA).

### 2.8. Statistical Analysis

Continuous variables were expressed as the median (Q1–Q3). Continuous variables were tested for normality using the Kolmogorov–Smirnov and Shapiro–Wilk tests. For comparing the differences in the groups, continuous variables with a normal distribution were compared by an independent samples t-test or a one-way ANOVA, with Bonferroni correction for multiple comparisons, while continuous variables with an abnormal distribution were compared by the Wilcoxon rank-sum test or Kruskal–Wallis test with Bonferroni correction for multiple comparisons. Categorical variables were expressed as the total numbers (percentages). The chi-square test or Fisher exact test was used to compare the categorical variables with Bonferroni correction if required by multiple comparisons. A statistical analysis was conducted with the SPSS version 23. GraphPad Prism 8 was used to evaluate the significance between the groups. A 2-tailed *p*-value < 0.05 was considered to be statistically significant.

## 3. Results

### 3.1. Clinical Characteristics

In this study, we enrolled 79 unrelated Chinese patients who had been clinically diagnosed and suspected to have BrS. Table 1 summarized the clinical and electrocardiographic characteristics of the 79 probands (69 males, 87.34%). The median age at diagnosis was 43 [36–54] years old. Of these, 32.91% showed spontaneous a type 1 BrS ECG. More than half of the patients (51.90%) experienced symptoms, including 7 patients (8.86%) with documented VT/VF, 28 patients (35.44%) with syncope, and 5 patients (6.33%) had a family history of unexplained SCD. Overall, 11 patients (13.92%) were implanted with an ICD. 

It is acknowledged that a type 1 BrS ECG is only a diagnostic pattern for BrS, while a those with a type 2 and 3 BrS ECG are suspected to have BrS. We grouped the patients by their types of BrS ECG. Compared with the type 2/3 BrS ECG group, the type 1 BrS ECG group displayed a longer QRS duration (109 [94–115] ms and 98 [89–106] ms, respectively; *p* = 0.004) and QTc interval (418 [405–438] ms and 409 [386–426] ms, respectively; *p* = 0.058) (Table 1). Patients with a type 1 BrS ECG tended to suffer more symptoms (65.38% and 45.28%, respectively; *p* = 0.093). Other electrocardiographic features, including the heart rate, P-wave duration, PR interval, all electrocardiographic axes, R-wave, and S-wave amplitude, did not differ between the type 1 ECG group and the type 2/3 ECG group (Table 1). 

Next, we grouped the patients according to the sex and symptoms. Males had a longer QRS duration (102 [93–112] ms and 88 [85–94] ms, respectively; *p* = 0.009), a higher R-wave amplitude in lead V5 (1.46 [1.21–1.83] mV and 0.77 [0.70–1.73] mV, respectively; *p* = 0.030), and a shorter QT and QTc interval than females (QT interval: 384 [363–400] ms and 411 [383–456] ms, respectively; *p* = 0.043) (QTc interval: 409 [390–425] ms and 450 [421–477] ms, respectively; *p* = 0.003) (Table S2). Similarly, compared with asymptomatic patients, symptomatic patients had a longer QT interval (396 [380–418] ms and 372 [360–389] ms, respectively; *p* = 0.001), QTc interval (420 [397–446] ms and 409 [384–422] ms, respectively; *p* = 0.045), and a higher rate of ICD implantations (24.39% and 2.63%, respectively; *p* = 0.005) (Table S3).

### 3.2. Genetic Testing and Screening Variants

**Figure 1:**
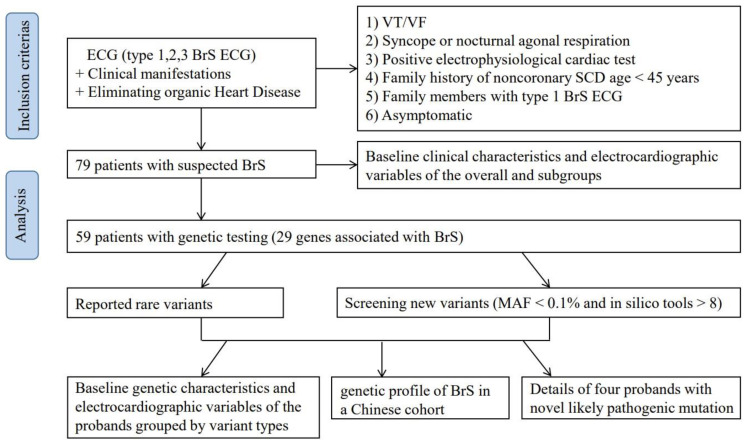
Flowchart of data collection and research process. BrS = Brugada syndrome; ECG = electrocardiogram; VT = ventricular tachycardia; VF = ventricular fibrillation; SCD = sudden cardiac death; and MAF = minor allele frequency.

**Figure 2:**
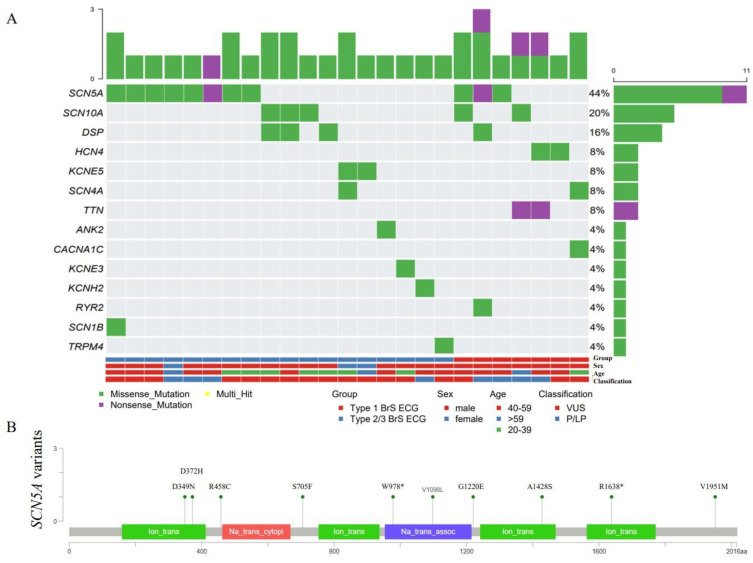
Waterfall plot and needle plot of the variants in genes associated with BrS. (**A**) A summary waterfall plot of variants in BrS-susceptibility genes. Each column represents 1 of the individuals, and each row represents a gene. The bar plot on the top shows the number of variants in each individual. The bar plot on the right shows the number of individuals containing a variant in each gene. (**B**) The variants needle plot of the *SCN5A* at the protein level across the BrS patients. A premature stop is symbolized by a black circle, whereas a green circle denotes an altered amino acid. BrS = Brugada syndrome; ECG = electrocardiogram; P/LP = patients with pathogenic or likely pathogenic mutations; and VUS = patients with uncertain significance variants.

Fifty-nine of the 79 probands underwent genetic testing, including 29 genes which are associated with BrS (Figure 1). In 25 probands, we identified 33 variants (42.37%) in 14 genes, including *HCN4*, *TTN*, *SCN5A*, *SCN1B*, *DSP*, *SCN10A*, *KCNH2*, *TRPM4*, *ANK2*, *RYR2*, *CACNA1C*, *SCN4A*, *KCNE5*, and *KCNE3* (Table S4). Of these, eight variants (13.56%) were classified as pathogenic/likely pathogenic (P/LP) according to the ACMG, and others were variants of an uncertain significance (VUS). *SCN5A* was the most common gene (Figure 2A), and 10 variants were found in 11 patients (18.64%), with 1 patient carrying 2 variants (Table 2). Most pathogenic mutations in *SCN5A* were located in the transmembrane regions (Figure 2B, Table 2).

### 3.3. Genetic Characteristics Analysis

As specific *SCN5A* mutations are linked to cardiac conduction disorders and electrocardiographic phenotypes, we analyzed the clinical characteristics of patients with or without *SCN5A* variants. We found that patients with *SCN5A* variants had a longer P-wave duration (112 [92–120] ms and 98 [87–102] ms, respectively; *p* = 0.038) and a higher RV1 + SV5 (0.76 [0.55–0.90] mV and 0.49 [0.31–0.72] mV, respectively; *p* = 0.037) than patients without *SCN5A* variants (Table S5). Moreover, *SCN5A* genetic-positive patients tended to present a longer QTc interval and larger T axis deviation than negative patients, though the results were not significant (Table S5).

Next, we divided these 59 patients into three groups based on the mutation types: (1) P/LP: patients with P/LP mutations (*n* = 8, 13.56%); (2) VUS: patients with uncertain significant variants (*n* = 17, 28.81%); and (3) negative: patients without variants (*n* = 34, 57.63%). Compared with the latter two groups, patients with P/LP mutations tended to have a longer QTc interval (432 [415–436] ms vs. 394 [384–434] ms vs. 411 [396–420] ms, respectively; *p* = 0.050) (Table 3). We further analyzed the clinical and electrocardiographic characteristics of the three subgroups in patients with a type 1 BrS ECG (Table S6). Patients with P/LP mutations showed prolonged QRS durations (120 [110–146] ms and 99 [91–112] ms, respectively; *p* = 0.009) (Figure S1A) and an R-wave axis deviation to the left (15 [–32–50] deg and 59 [42–64] deg, respectively; *p* = 0.027) (Figure S1B) than patients without variants. In addition, patients carrying P/LP mutations showed a higher S-wave amplitude in lead V5 (0.68 [0.26–1.17] mV and 0.20 [0.06–0.47] mV, respectively; *p* = 0.037) (Figure S1C), a decreased S-wave amplitude in lead V1 (0.24 [0.03–0.64] mV and 1.00 [0.59–1.21] mV, respectively; *p* = 0.014) (Figure S1D), and a lower RV5 + SV1 (1.49 [1.19–1.78] mV and 2.53 [1.87–2.90] mV, respectively; *p* = 0.063) (Figure S1E) compared with non-carriers. However, in the type 2/3 BrS ECG group, we did not find any significant difference (Table S6).

### 3.4. Clinical and Genetic Features of Four Probands Carrying Novel P/LP Mutations

**Figure 3:**
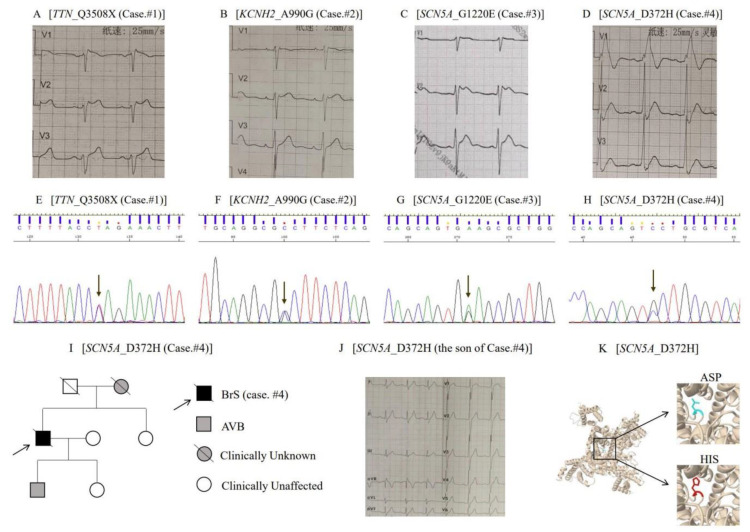
Representative cases of BrS with novel likely pathogenic or pathogenic mutations. (**A**–**D**) Each panel shows an electrocardiogram of BrS cases carrying novel mutations. (**E**–**H**) All 4 novel mutations of BrS identified in the study were validated by Sanger sequencing. (**I**) Pedigree of a representative family (Case 4) with *SCN5A* likely pathogenic mutation. (**J**) An electrocardiogram of the son of case 4. (**K**) The schematic structure and details of D372H in *SCN5A* was predicted with Missense3D. BrS = Brugada syndrome; and AVB = atrioventricular block.

In our cohort, we screened eight novel variants, and four of them were classified as P/LP mutations: one was a titin (*TTN*) mutation, p.Q3508X (c.10522C > T); one was a *KCNH2* mutation, p.A990G (c.2969C > G); and two were *SCN5A* mutations, p.G1220E (c.3659G > A) and p.D372H (c.1114G > C) (Table S4). All four mutations were absent from the controls in ExAc and gnomAD and were predicted deleterious with more than eight bioinformatic tools (Table S4). The details of these mutation carriers were as follows:

Case 1: The first mutation was p.Q3508X, a nonsense mutation in *TTN* (c.10522C > T) (Figure 3E, Table S4). The carrier was a 66-year-old male, diagnosed with a right brachial plexus injury without any other symptoms. His 12-lead ECG exhibited a coved-type ST elevation in the V1 lead and a saddleback-type ST elevation in the V2 lead (Figure 3A). The novel c.10522C > T variant in the *TTN* gene was classified as pathogenic according to the ACMG.

Case 2: The proband, a 46-year-old man, was admitted to the hospital for a recurrent fever, which induced a type 2 BrS ECG in the V2 lead (Figure 3B). He was asymptomatic and did not receive a drug provocation test, but his nephew died suddenly at night when he was 16. We identified a heterozygous p.A990G (c.2969C > G) in the *KCNH2* by genetic screening (Figure 3F, Table S4). However, more detailed clinical and genetic information about his relatives was not available. The novel c.2969C > G variant in the *KCNH2* gene was classified as being likely pathogenic according to the ACMG.

Case 3: The third mutation, a heterozygous p.G1220E (c.3659G > A) of *SCN5A*, was identified in an asymptomatic 40-year-old man (Figure 3G, Table S4). His 12-lead ECG showed a coved-type ST elevation in the V1 lead and a saddleback-type ST elevation in the V2 lead (Figure 3C). The novel c.3656G > A variant in the *SCN5A* gene was classified as being likely pathogenic according to the ACMG.

Case 4: A heterozygous p.D372H (c.1114G > C) of the *SCN5A* was identified in a 48-year-old man (Figure 3H, Table S4) who was admitted to the hospital for sudden syncope. This patient had sleep apnea syndrome and a paroxysmal II degree I atrioventricular block. His 12-lead ECG revealed a significant coved-type ST elevation (Figure 3D). His father died at 81 years old without any cardiovascular disease. His mother had a history of syncope and died suddenly at the age of 50 due to unexplained heart discomfort (Figure 3I). We also performed genetic testing on his sister, son, and daughter. The patient’s sister and daughter had no gene variants or symptoms (Figure 3I). Only his son carried the same variant, suffering a first-degree atrioventricular block, and a slight ST elevation (Figure 3J). The son showed a longer P-wave duration (116 ms), QRS duration (152 ms), T-wave duration (200 ms), and PR interval (212 ms). Furthermore, we used Missense3D to detect the structural change due to the substitution of the amino acid. The Missense3D tool predicted that this substitution replaced a buried negative-charged residue (ASP, RSA 1.8%) with a positive-charged residue (HIS) (Figure 3K). Thus, the novel c.1114G > C variant in the *SCN5A* gene was classified as being likely pathogenic according to the ACMG.

## 4. Discussion

The main findings in this study were as follows: (1) we reported the spectrum of genetic variations in 29 BrS-susceptibility genes in a suspected BrS cohort from China; (2) we identified one novel mutation in *TTN*, one novel mutation in *KCNH2*, and two novel mutations in *SCN5A*; and (3) we found that the electrocardiographic axes should also be considered when predicting the risk of arrhythmias in BrS patients.

BrS can be found all over the world, but is more prevalent in Asia, including a higher prevalence of a type 1 BrS ECG (0–0.36%) and a type 2/3 BrS ECG (0.12–2.23%) [22]. The risk prediction for an SCD is a key issue in the management of patients with BrS. It is well known that males [23,24], a spontaneous type 1 BrS ECG [25], the presence of symptoms (such as arrhythmic syncope and documented VT/VF) [26], *SCN5A* mutations [27], and various electrocardiographic markers [28] are shown to be significant predictors of an SCD. To date, two principal hypotheses have been proposed: the repolarization hypothesis and the depolarization hypothesis [29], which may together lead to BrS under the influence of other factors such as sex, age, genetic background, and a fever.

In this study, we enrolled a Chinese cohort suspected of having BrS and carried out multiple subgroup analyses to compare their clinical and electrocardiographic characteristics. Depolarization abnormalities were prominent in our study, including a prolonged QRS duration, P-wave duration, and a decreased QRS amplitude. The repolarization abnormalities were mainly exhibited as a QT or QTc interval prolongation. The details are described as follows:

First, we compared the common ECG parameters in patients with a spontaneous type 1 BrS ECG and patients with a type 2/3 BrS ECG. Consistent with the previous report [25], compared with the type 2/3 BrS ECG group, patients with a type 1 BrS ECG exhibited a prolonged QRS duration and QTc interval. A prolonged QRS is attributed to sodium current dysfunction in the conduction system and specifically in the His-Purkinje system, which is linked to a poor prognosis, as has been confirmed by several studies [30,31]. A QTc prolongation, reflecting a delayed cellular repolarization, has been associated with an increased risk of VT/VF and an SCD in BrS [32,33]. The results indicated that the association between a spontaneous type 1 BrS ECG and a delayed activation [34] could lead to a more severe phenotype, as we showed. In addition, we found that males had a longer QRS duration than females, whereas females exhibited longer QT and QTc durations. Similarly [33], the symptomatic group displayed longer QT and QTc intervals and suffered more arrhythmic events with a higher rate of ICD implantation. The above results indicated that males, patients with a spontaneous type1 BrS ECG, and symptoms likely had a greater risk of an SCD.

Next, we explored the differences in ECG features between groups of 59 patients divided by their mutation types. Consistent with previous studies [35,36], we observed a greater P-wave duration and QTc interval in the *SCN5A* variant carriers than in the non-carriers. It has been confirmed that sodium channels dysfunction or downregulatory can impair atrial and ventricular conduction [37]. In addition, we found a higher RV1 + SV5 in patients with *SCN5A* variants. RV1 + SV5 indicates right ventricular hypertrophy if the value is greater than 1.05 mV. While the values were within the normal ranges in our patients, carriers tended to show a delayed right ventricular conduction, as reported in [38]. Moreover, we observed that patients with *SCN5A* variants had a larger T axis deviation to the right. The T axis, an index of primary repolarization abnormality, could be affected by an action potential duration shortening or prolongation in any ventricular region. Thus, the T axis has been reported as a strong predictor of fatal and nonfatal cardiac events [13,39], even when the value is greater than 45° [40]. In our study, the larger T axis deviation to the right in patients with *SCN5A* variants might also suggest a higher risk of arrhythmic events. 

Without any significant difference among three subgroups of all patients, we further explored the electrocardiographic characteristics in patients with a spontaneous type 1 ECG and found marked depolarization abnormalities of the right ventricle in patients with pathogenic mutations, including an increased QRS duration, decreased S-wave amplitude in lead V1, and decreased RV5 + SV1. Moreover, patients with P/LP mutations showed a prominent deviation to the left of the R axis. This is an index for cardiac conduction, which had been reported with a left shift in males [41].

Here, we described the genetic profile in our suspected BrS cohort. The top three genes were *SCN5A* (20%), *SCN10A* (8%), and *DSP* (7%). The *SCN5A* gene encodes the α-subunit of the cardiac voltage-gated sodium channel (Nav1.5) protein, which consists of four homologous domains (DI-DIV) that are connected by intracellular linkers. Each domain contains six transmembrane-spanning segments (S1–S6). We identified five pathogenic mutations in *SCN5A*, accounting for 8% of BrS patients, and four of them were localized to the transmembrane and pore-forming domains, similar to a previous report [10,14]. *SCN5A* is responsible for initiating the cardiac action potential. Pathogenic mutations result in a sodium channel dysfunction, which slows the impulse conduction throughout the myocardium [42]. The burden of a rare variation in *SCN10A* varied across the regions, from 2.5% in Japanese probands [43] to 16.7% in American probands [44]. Different from the previous study [45], we found that fewer patients carried variants in *CACNA1C* (2%), whereas more carried variants in *DSP* (7%) and *HCN4* (3%). In addition, we found two nonsense mutations in *TTN* (3%) and classified them as pathogenic mutations. *TTN* is associated with several diseases, including inherited arrhythmias. Currently, only one frameshift mutation in *TTN* has been reported, which is likely to be pathogenic for BrS [46]. 

In summary, we reported four novel P/LP mutations in our cohort. However, it is important to determine the clinical significance by functional studies. All the results showed that Chines BrS patients had a different spectrum of genetic variations.

## 5. Study Limitations

Several limitations of the study should be noted. First, this was a retrospective study, and we did not perform drug challenges for patients with a type 2/3 BrS ECG. This might hamper their clinical diagnosis and might mask some significant differences between the groups. Second, most patients underwent target sequencing rather than WES. As we all know, the genetic background of more than 60% of BrS patients remains unclear, thus it is important to screen novel disease-causing genes for BrS. Third, we have to acknowledge that the study scale is relatively small, and multiple subgroup analyses may lead to an over-interpretation of the results. Finally, we did not conduct functional studies to determine the pathogenicity of the novel variants.

## 6. Conclusions

In this study, we compared the clinical and electrophysiologic characteristics of suspected Chinese BrS patients grouped by the types of BrS ECG, sex, symptoms, and genetic features. Furthermore, we identified four novel pathogenic mutations in the cohort and showed a representative pedigree. Our results suggest that BrS patients have significant depolarization and repolarization abnormalities, which may increase the risk of arrhythmic events and SCD.

## Figures and Tables

**Table 1:** Clinical and ECG characteristics of the included subjects.

Feature	Overall (*n* = 79)	Type 1 BrS ECG (*n* = 26)	Type 2/3 BrS ECG (*n* = 53)	*p*-Value
Male (*n*, %)	69 (87.34%)	25 (96.15%)	44 (83.02%)	0.197
Age at diagnosis (years)	43 (36–54)	47 (32–52)	42 (36–54)	0.697
Spontaneous Type 1 ECG (*n*, %)	26 (32.91%)	26	0	—
Symptomatic patients (*n*, %)	41 (51.90%)	17 (65.38%)	24 (45.28%)	0.093
Documented VT/VF (*n*, %)	7 (8.86%)	2 (7.69%)	5 (9.43%)	1.000
Syncope (*n*, %)	28 (35.44%)	10 (38.46%)	18 (33.96%)	0.694
Family history of SCD (*n*, %)	5 (6.33%)	2 (7.69%)	3 (5.66%)	1.000
ICD (*n*, %)	11 (13.92%)	5 (19.23%)	6 (11.32%)	0.543
Heart rate (bpm)	70 (62–76)	71 (65–75)	70 (62–77)	0.688
P-wave duration (ms)	100 (86–104)	100 (80–104)	98 (87–107)	0.810
QRS duration (ms)	100 (90–109)	109 (94–115)	98 (89–106)	0.004 *
T-wave duration (ms)	162 (120–200)	180 (150–200)	160 (110–200)	0.172
PR interval (ms)	162 (142–180)	166 (144–189)	160 (136–177)	0.244
QT interval (ms)	388 (363–407)	389 (368–402)	388 (360–414)	0.770
QTc interval (ms)	411 (390–432)	418 (405–438)	409 (386–426)	0.058
P-wave axis (deg)	57 (41–67)	49 (40–66)	61 (40–71)	0.612
R-wave axis (deg)	48 (21–64)	48 (32–62)	49 (19–69)	0.972
QRS axis (deg)	47 (15–69)	53 (14–77)	44 (16–69)	0.342
T-wave axis (deg)	50 (29–66)	48 (28–66)	52 (32–68)	0.437
R-wave Amplitude in lead V1 (mV)	0.17 (0.10–0.32)	0.25 (0.05–0.36)	0.15 (0.10–0.32)	0.758
R-wave Amplitude in lead V5 (mV)	1.46 (1.13–1.80)	1.43 (1.13–1.78)	1.46 (1.11–1.81)	0.946
S-wave Amplitude in lead V1 (mV)	0.66 (0.39–1.06)	0.60 (0.35–1.00)	0.66 (0.42–1.09)	0.555
S-wave Amplitude in lead V5 (mV)	0.30 (0.10–0.50)	0.30 (0.10–0.66)	0.30 (0.11–0.48)	0.500
RV5 + SV1 (mV)	2.18 (1.60–2.65)	2.20 (1.59–2.62)	2.15 (1.65–2.67)	0.594
RV1 + SV5 (mV)	0.55 (0.38–0.80)	0.58 (0.42–0.86)	0.50 (0.37–0.80)	0.431

For categorical variables, the data were presented as number (%). For continuous variables, the data were expressed as median (Q1–Q3). * *p* < 0.05. BrS = Brugada syndrome; ECG = electrocardiogram; VT = ventricular tachycardia; VF = ventricular fibrillation; SCD = sudden cardiac death; ICD = implantable cardioverter defibrillator; and deg = degree.

**Table 2:** Clinical and genetic characteristics of *SCN5A* variant carriers.

Age at Diagnosis	Sex	Symptom	Type of BrS ECG	Amino Acid Change	rs Number	Nucleotide Change	Variant Type	Exon	Location	ACMG
43	M	(+)	1	p.S705F	rs199473148	c.2114C > T	Missense	14	DI-DII	VUS
54; 53	M; M	(+); (−)	2; 2	p.V1951M	rs41315493	c.5851G > A	Missense	28	C-terminus	VUS
66	F	(−)	2	p.A1428S	rs200034939	c.4282G > T	Missense	24	DIII-S5/S6	LP
47	M	(+)	1	p.D349N	rs779687673	c.1045G > A	Missense	9	DI-S5/S6	VUS
40	M	(−)	1	p.G1220E	.	c.3659G > A	Missense	20	DIII-S1	LP
59	M	(−)	3	p.W978X	.	c.2933G > A	Nonsense	17	DII-DIII	P
30; 34	M; M	(−); (−)	2; 2	p.V1098L	rs199473191	c.3292G > T	Missense	18	DII-DIII	VUS
34	M	(−)	2	p.R458C	rs752130196	c.1372C > T	Missense	11	DI-DII	VUS
48	M	(+)	1	p.D372H	.	c.1114G > C	Missense	9	DI-S5/S6 (Pore)	LP
41	M	(+)	1	p.R1638X	rs761505217	c.4912C > T	Nonsense	28	DIV-S4/S5	P

BrS = Brugada syndrome; ECG = electrocardiogram; ACMG = American College of Medical Genetics and Genomics; M = Male; F = Female; “+” = symptomatic; “−“ = asymptomatic; 1 = type 1 BrS ECG; 2 = type 2 BrS ECG; 3 = type 3 BrS ECG; VUS = variants of uncertain significance; LP = likely pathogenic; and P = pathogenic.

**Table 3:** Clinical and ECG characteristics of the included subjects classified by the mutation pathogenicity.

Feature	P/LP (*n* = 8)	VUS (*n* = 17)	Negative (*n* = 34)	*p*-Value
Male (*n*, %)	7 (87.50%)	15 (88.24%)	28 (82.35%)	0.880
Age at diagnosis (years)	49 (42–64)	40 (31–49)	44 (36–55)	0.091
Spontaneous Type 1 ECG (*n*, %)	4 (50.00%)	3 (17.65%)	12 (35.29%)	0.237
Symptomatic patients (*n*, %)	3 (37.50%)	8 (47.06%)	22 (64.71%)	0.243
Documented VT/VF (*n*, %)	0 (0.00%)	1 (5.88%)	2 (5.88%)	1.000
Syncope (*n*, %)	2 (25.00%)	4 (23.53%)	15 (44.12%)	0.319
ICD (*n*, %)	0 (0.00%)	3 (17.65%)	2 (5.88%)	0.395
Heart rate (bpm)	74 (73–81)	70 (62–74)	69 (61–76)	0.097
P-wave duration (ms)	113 (98–119)	98 (88–103)	98 (86–104)	0.145
QRS duration (ms)	105 (97–123)	96 (90–111)	100 (88–108)	0.074
T-wave duration (ms)	180 (105–200)	180 (150–210)	159 (100–196)	0.200
PR interval (ms)	173 (161–203)	164 (154–186)	153 (128–175)	0.107
QT interval (ms)	385 (370–407)	390 (366–417)	385 (360–400)	0.862
QTc interval (ms)	432 (415–436)	394 (384–434)	411 (396–420)	0.050
P-wave axis (deg)	49 (41–70)	61 (48–69)	59 (39–66)	0.727
R-wave axis (deg)	24 (-15–79)	42 (21–63)	49 (25–63)	0.403
QRS axis (deg)	24 (-19–98)	34 (12–73)	48 (30–64)	0.640
T-wave axis (deg)	41 (4–72)	53 (35–81)	55 (29–66)	0.371
R-wave Amplitude in lead V1 (mV)	0.23 (0.08–0.34)	0.12 (0.08–0.26)	0.15 (0.09–0.36)	0.516
R-wave Amplitude in lead V5 (mV)	1.22 (1.02–1.49)	1.34 (1.04–1.79)	1.47 (0.99–1.85)	0.510
S-wave Amplitude in lead V1 (mV)	0.58 (0.17–0.82)	0.72 (0.48–1.05)	0.87 (0.39–1.13)	0.689
S-wave Amplitude in lead V5 (mV)	0.37 (0.26–0.91)	0.30 (0.17–0.57)	0.28 (0.10–0.46)	0.220
RV5 + SV1 (mV)	1.72 (1.19–2.22)	2.15 (1.67–2.93)	2.32 (1.68–2.90)	0.445
RV1 + SV5 (mV)	0.72 (0.48–1.06)	0.58 (0.30–0.81)	0.50 (0.37–0.75)	0.336

For categorical variables, the data were presented as number (%). For continuous variables, the data were expressed as median (Q1–Q3). ECG = electrocardiogram; VT = ventricular tachycardia; VF = ventricular fibrillation; ICD = implantable cardioverter defibrillator; deg = degree. P/LP = patients with pathogenic or likely pathogenic mutations; VUS = patients with uncertain significance variants; and negative = patients without variants.

## Data Availability

The data of this study are available on request from the corresponding authors.

## Supplementary Materials

The following supporting information can be downloaded at: https://www.mdpi.com/article/10.3390/jcdd9110369/s1, Figure S1: Comparision of electrocardiographic characteristics between P/LP and Negative groups in patients with type 1 BrS ECG; Table S1: PCR primers for Sanger sequencing for all rare variants; Table S2: Clinical and ECG characteristics of the included subjects classified by sex; Table S3: Clinical and ECG characteristics of the included subjects classified by symptoms; Table S4: Rare variants of the included subjects; Table S5: Clinical and ECG characteristics of the included subjects classified by *SCN5A* variants; Table S6: Clinical and ECG characteristics of different ECG groups classified by mutation pathogenicity.
