# Supplementary material for: Genetic Profile and Clinical Characteristics of Brugada Syndrome in the Chinese Population
Source: J Cardiovasc Dev Dis. 2022 Oct 28;9(11):369. doi: 10.3390/jcdd9110369 (PMC9699371; doi:10.3390/jcdd9110369)
Supplement: Supplementary file 1 [file jcdd-09-00369-s001.zip › jcdd-1956317-supplementary.pdf]

Supplementary Materials for

Genetic profile and clinical characteristics of Brugada Syndrome in the Chinese population

Linlin Wang[1,2], Yanghui Chen[1,2], Yang Sun[1,2], Man Huang[1,2], Haoran Wei[1,2], Hao Liu[1,2], Ke Xu[1,2], Xiuli Song[1,2,3], Peng Chen[1,2], Lun Tan[1,2], Jin Huang[1,2], Zongzhe Li[1,2], Rui Li[1,2], Ting Yu[1,2], Fei Ma[1,2], Hu Ding[1,2], Yan Wang[1,2], Daowen Wang[1,2,3], Hong Wang[1,2,3*], Chunxia Zhao[1,2*]

Figure S1. Comparision of electrocardiographic characteristics between P/LP and Negative groups in patients with type 1 BrS ECG.

Table S1. PCR primers for Sanger sequencing for all rare variants.

Table S2. Clinical and ECG characteristics of the included subjects classified by sex.

Table S3. Clinical and ECG characteristics of the included subjects classified by symptoms.

Table S4. Rare variants of the included subjects.

Table S5. Clinical and ECG characteristics of the included subjects classified by *SCN5A* variants.

Table S6. Clinical and ECG characteristics of different ECG groups classified by mutation pathogenicity.

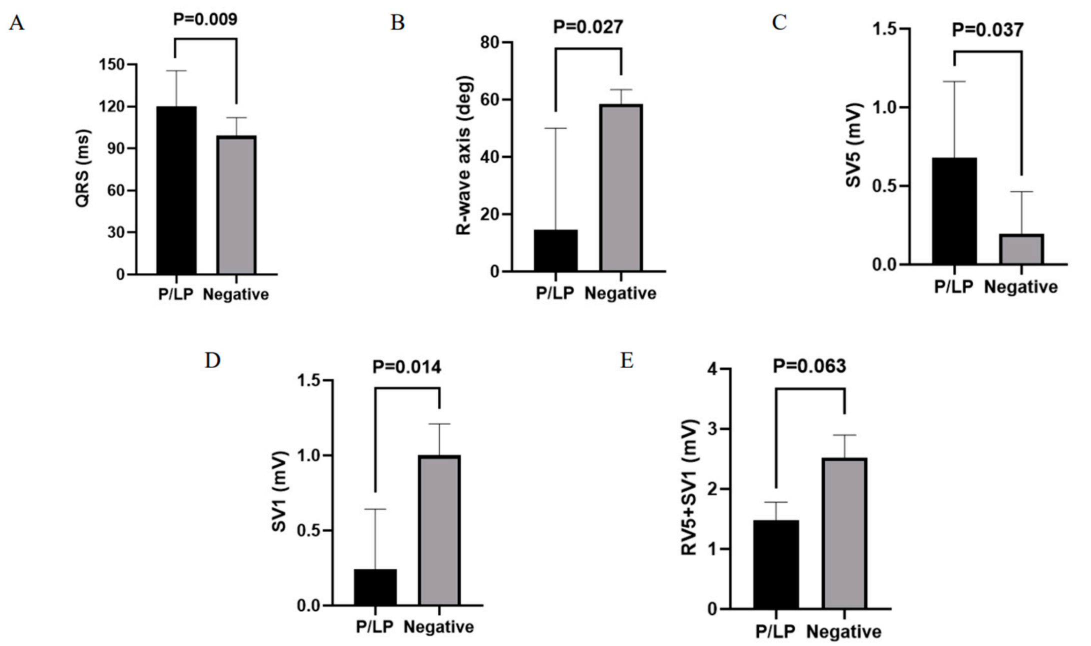

Figure S1. Comparision of electrocardiographic characteristics between P/LP and Negative groups in patients with type 1 BrS ECG.

P/LP = patients with pathogenic or likely pathogenic mutations; Negative = patients without variants; deg = degree; BrS = brugada syndrome; ECG = electrocardiogram.

Table S1. PCR primers for Sanger sequencing for all rare variants.

Gene	Amino Acid Change	Primers
ANK2	L2247I	forward: 5'-TCCTTTCAACACAACATTTCCAC-3'; reverse: 5'-TGCAATCATCTTGTCTTTTAGGG-3'
CACNA1C	G1700S	forward: 5'-TAGGGACCACCATCCTAGACGT-3'; reverse: 5'-CCTCCACTGTCTCCTGAGGGT-3'
DSP	R1341H	forward: 5'-CCAAAAATCTTAGAAACCAGCTTG-3'; reverse: 5'-TCTGTGGTCTGGGTTAGAGTGTTC-3'
	R1725W	forward: 5'-GGAGCAGGCATCCATTGTTAA-3'; reverse: 5'-CCAAACACAGGCTACCCAGAG-3'
	D2155G	forward: 5'-GGTGCAGGATCTATCGCTGG-3'; reverse: 5'-CCTTAATTCTCTCACCAACCTCG-3'
	S2349C	forward: 5'-TTCAAGCTGCATAGCAGGCA-3'; reverse: 5'-AGGCAGAAGACAGAGCCCTGT-3'
HCN4	S70L	forward: 5'-CGCAAGCGGCTCTACAGC-3'; reverse: 5'-GCCTCCCTCCACTTTGATAGC-3'
	A195V	forward: 5'-GGGCAAGTCCAGCACGAA-3'; reverse: 5'-TGGCCCCTTACCTGAAGTCA-3'
KCNE3	R81C	forward: 5'-GCCACCAGAGCTTCTACCGA-3'; reverse: 5'-TTCATGGGCTCCCACTGTTT-3'
KCNE5	Y81H	forward:5'-TGAACTGCAGCGAGAGCCA-3'; reverse:5'-GGGCTGTGGTTTTAGACCCG-3'
KCNH2	A990G	forward:5'-GAGAAGAGCAGCGACACTTGC-3'; reverse:5'-TCCCTCTACCAGACAACACCG-3'
RYR2	V3979L	forward: 5'-TATTTGGCCCTCCTTTAGTTCC-3'; reverse: 5'-TCCGAAACCCCCACTGATTA-3'
SCN10A	G98R	forward:5'-GCCCTTCTTGCTCATAAGCCT-3'; reverse:5'-GAGGCAATCGTGCAAAGGAT-3'
	P572L	forward: 5'-AGGTAGAGGGAAGTGTTGGGC-3'; reverse: 5'-CTGAGGCAGTGATGTTGAGTGG-3'
	T872M	forward: 5'-TCATCGGAAACTCAGTGGGG-3'; reverse: 5'-TCCCAGTAAACCCATGAGTAAGC-3'
	L1184F	forward:5'-GCTGGGTCCTTCCCTATCAGT-3'; reverse:5'-CCCACTGAGCCACTCACATTC-3'
	P1918L	forward:5'-CGCTCCATGGCACTCTCTAAC-3'; reverse:5'-TCCAGGCTGGAGTGTTCTCAC-3'
SCN1B	C262Y	forward:5'-GCAGGCAGTGGACAGGACA-3'; reverse:5'-AACTCTGCTCTAGGTGGCACG-3'
SCN4A	F103V	forward: 5'-GGAGTCACTGGCAGCCATAGA-3'; reverse: 5'-GAAAGACAAGAGCAGCACCACA-3'

	L556F	forward:5'-TGCTAGAGGGTTGAGATGTGGA-3';<br>reverse:5'-CCCATATGCTGGGAAAATAACC-3'
SCN5A	D349N	forward: 5'-ACTCCTCATGGGGTAGATGGG-3';<br>reverse: 5'-AAGGGACTGGTACTTCATCACCA-3'
	D372H	forward:5'-TGAGCCAGGCTTGTGTGCT-3';<br>reverse:5'-GGCAGAATGTTCCACCCAAC-3'
	R458C	forward: 5'-TCATCTACACCCACCTCCCAC-3';<br>reverse: 5'-TCCCCTACTCTAAGGAAGGCC-3'
	S705F	forward:5'-TTCCAGATTAAGGAGCCAGGAC-3';<br>reverse:5'-GCAGCATCTCCTCGAATTCAC-3'
	W978X	forward: 5'-GCCAAACCTTCCACATTCAATA-3';<br>reverse: 5'-CACCTGCTTGCTGGACTCCT-3'
	V1098L	forward:5'-AAGACAGCTGGAGGCAATGC-3';<br>reverse:5'-GCATCAGAGTTTGGGACCAGA-3'
	G1220E	forward:5'-GCCAGGCTGTTCACCCACT-3';<br>reverse:5'-TGCACTGTGACAGCTTGACAGA-3’
	A1428S	forward: 5'-GAAATGGAAATGAGGGTGGATC-3';<br>reverse: 5'-CCATCCTCCTGGGGTAAAAGT-3'
	R1638X	forward: 5'-CAGATGCAGACATGGAGACTCAA-3';<br>reverse: 5'-GACCACGATGAGGAAGGAGATG-3'
	V1951M	forward: 5'-CAAGCCCAACCAGATAAGCC-3';<br>reverse: 5'-AGGCCCATTTCTTACTCCCAA-3'
TRPM4	S981W	forward:5'-GGCATGTTCTCGAATCACCAG-3';<br>reverse:5'-GTTCGTACTCGCGGATGTGTC-3'
TTN	Q3508X	forward:5'-ATGGTGTTGCGTTTATTCGAAC-3';<br>reverse:5'-CTCCACATTTTCATGTGTCCGT-3'
	R4705X	forward: 5'-TCCTAGCCCTGAAACGTTACAA-3';<br>reverse: 5'-GGAAGCACTGACTCAGGGAGAG-3'

Table S2. Clinical and ECG characteristics of the included subjects classified by sex.

Feature	Male (n = 69)	Female (n = 10)	P-value
Age at diagnosis (years)	43 (36-54)	39 (28-62)	0.665
Spontaneous Type 1 ECG (n, %)	25 (36.23%)	1 (10.00%)	0.197
Symptomatic patients (n, %)	34 (49.28%)	7 (70.00%)	0.375
Documented VT/VF (n, %)	6 (8.70%)	1 (10.00%)	1.000
Syncope (n, %)	23 (33.33%)	5 (50.00%)	0.499
Family history of SCD (n, %)	4 (5.80%)	1 (10.00%)	0.501
ICD (n, %)	8 (11.59%)	3 (30.00%)	0.279
Heart rate (bpm)	70 (62-76)	71 (62-85)	0.516
P-wave duration (ms)	100 (87-107)	95 (78-104)	0.314
QRS duration (ms)	102 (93-112)	88 (85-94)	0.009*
T-wave duration (ms)	172 (120-200)	130 (100-200)	0.567
PR interval (ms)	164 (145-180)	136 (121-176)	0.146
QT interval (ms)	384 (363-400)	411 (383-456)	0.043*
QTc interval (ms)	409 (390-425)	450 (421-477)	0.003*
P-wave axis (deg)	55 (37-67)	64 (47-78)	0.337
R-wave axis (deg)	48 (21-64)	50 (23-74)	0.762
QRS axis (deg)	47 (12-70)	49 (37-73)	0.606
T-wave axis (deg)	52 (30-67)	48 (8-66)	0.555
R-wave Amplitude in lead V1 (mV)	0.20 (0.09-0.32)	0.12 (0.10-0.49)	0.953
R-wave Amplitude in lead V5 (mV)	1.46 (1.21-1.83)	0.77 (0.70-1.73)	0.030*
S-wave Amplitude in lead V1 (mV)	0.66 (0.39-1.03)	0.74 (0.46-1.53)	0.115
S-wave Amplitude in lead V5 (mV)	0.30 (0.10-0.53)	0.20 (0.10-0.35)	0.268
RV5+SV1(mV)	2.20 (1.72-2.63)	1.80 (1.22-3.19)	0.345
RV1+SV5(mV)	0.55 (0.40-0.81)	0.50 (0.25-0.83)	0.560

For categorical variables, the data were presented as number (%). For continuous variables, the data were expressed as median (Q1–Q3). *P < 0.05

ECG = electrocardiogram; VT = ventricular tachycardia; VF = ventricular fibrillation; SCD = sudden cardiac death; ICD = implantable cardioverter defibrillator; deg = degree.

Table S3. Clinical and ECG characteristics of the included subjects classified by symptoms.

Feature	Symptomatic patients (n = 41)	Asymptomatic patients (n = 38)	P-value
Male (n, %)	34 (82.93%)	35 (92.11%)	0.375
Age at diagnosis (years)	43 (37-52)	42 (34-57)	0.862
Spontaneous Type 1 ECG (n, %)	17 (41.46%)	9 (23.68%)	0.093
ICD (n, %)	10 (24.39%)	1 (2.63%)	0.005*
Heart rate (bpm)	67 (61-76)	72 (63-77)	0.177
P-wave duration (ms)	100 (84-107)	98 (89-105)	0.836
QRS duration (ms)	100 (90-109)	100 (92-112)	0.914
T-wave duration (ms)	178 (110-200)	160 (120-200)	0.801
PR interval (ms)	166 (137-186)	161 (145-177)	0.735
QT interval (ms)	396 (380-418)	372 (360-389)	0.001*
QTc interval (ms)	420 (397-446)	409 (384-422)	0.045*
P-wave axis (deg)	58 (33-66)	52 (43-75)	0.602
R-wave axis (deg)	45 (19-62)	51 (30-70)	0.167
QRS axis (deg)	43 (12-60)	51 (19-77)	0.213
T-wave axis (deg)	48 (21-66)	56 (46-69)	0.043*
R-wave Amplitude in lead V1 (mV)	0.15 (0.05-0.35)	0.19 (0.10-0.30)	0.720
R-wave Amplitude in lead V5 (mV)	1.39 (1.10-1.68)	1.48 (1.18-1.82)	0.261
S-wave Amplitude in lead V1 (mV)	0.68 (0.39-1.00)	0.58 (0.38-1.09)	0.753
S-wave Amplitude in lead V5 (mV)	0.30 (0.10-0.50)	0.30 (0.10-0.58)	0.933
RV5+SV1(mV)	2.18 (1.55-2.68)	2.18 (1.80-2.56)	0.648
RV1+SV5(mV)	0.55 (0.38-0.77)	0.50 (0.36-0.86)	0.883

For categorical variables, the data were presented as number (%). For continuous variables, the data were expressed as median (Q1–Q3). *$P < 0.05$

ECG = electrocardiogram; ICD = implantable cardioverter defibrillator; deg = degree.

Table S4. Rare variants of the included subjects.

Gene	Transcript	Nucleotide Change	Amino Acid Change	Variant Type	ExAC_EAS	AF_eas	SIFT	Polyphen2	LRT	MutationTaster	MutationAssessor	FATHMM	PROVEAN	MetaSVM	MetaLR	M-CAP	REVEL	CADD	fathmm-MKL	GERP	ACMG	Reported/Novel
HCN4	NM_005477	c.209C > T	p.S70L	missense	0.0089	0.0005	T	B	.	D	N	D	N	D	D	D	0.255	22.6	D	3.21	VUS	Reported
TTN	NM_133379	c.14113C > T	p.R4705X	nonsense	0.0004	0.0003	.	.	.	D	.	.	.	.	.	.	.	10.59	N	3.82	P	Reported
SCN5A	NM_198056	c.2114C > T	p.S705F	missense	0.0008	0.0006	D	B	N	D	L	D	D	D	D	D	0.53	22.9	D	4.9	VUS	Reported
SCN1B	NM_199037	c.785G > A	p.C262Y	missense	0	0.0019	D	B	.	N	.	D	N	T	D	D	0.086	10.19	N	-0.381	VUS	Reported
DSP	NM_004415	c.4022G > A	p.R1341H	missense	0.0006	0.0003	D	B	D	D	L	D	D	D	D	D	0.458	24.5	D	5.51	VUS	Reported
SCN	NM_0	c.29	p.G	mis	.	.	D	P	D	D	M	D	D	D	D	D	0.5	23	D	3.	V	No

10A	01293306	2G>A	98R	sense													77	.7		64	US	vel
SCN5A	NM_198056	c.5851G>A	p.V1951M	missense	0.003	0.0007	T	B	N	N	N	D	N	T	T	D	0.521	7.984	N	-0.527	VUS	Reported
KCNH2	NM_000238	c.2969C>G	p.A990G	missense	.	.	T	D	D	D	L	D	N	D	D	D	0.642	23.7	D	4.98	LP	Novel
TRPM4	NM_001195227	c.2942C>G	p.S981W	missense	0	0	D	D	N	D	M	T	D	T	T	D	0.17	28.4	D	4.57	VUS	Reported
SCN5A	NM_198056	c.4282G>T	p.A1428S	missense	0.0003	0.0004	D	D	D	D	M	D	D	D	D	D	0.937	31	D	4.1	LP	Reported
SCN5A	NM_198056	c.1045G>A	p.D349N	missense	0	0	T	B	D	D	L	D	N	D	D	D	0.442	22.7	D	4.73	VUS	Reported
SCN10A	NM_001293306	c.3552G>T	p.L1184F	missense	0.0012	0.0014	D	B	N	N	L	D	N	D	D	D	0.376	21.5	D	3.28	VUS	Reported
SCN5A	NM_198056	c.3659G>A	p.G1220E	missense	.	0	D	D	D	D	H	D	D	D	D	D	0.899	28.1	D	4.21	LP	Novel
SCN5A	NM_198056	c.2933G	p.W978	nonsen	.	.	.	.	N	A	.	.	.	.	.	.	.	39	D	4.46	P	Reporte

		>A	X	se																		d
SCN5A	NM_198056	c.3292G>T	p.V1098L	missense	0.002	0.002	T	B	N	D	L	D	N	D	D	D	0.387	20.1	D	4.3	VUS	Reported
SCN5A	NM_198056	c.1372C>T	p.R458C	missense	0	5.59E-05	T	P	N	D	N	D	D	D	D	D	0.561	23.8	D	4.36	VUS	Reported
SCN10A	NM_001293306	c.5753C>T	p.P1918L	missense	.	0	D	D	D	D	M	D	D	D	D	D	0.761	25.2	D	5.38	VUS	Reported
TTN	NM_133379	c.10522C > T	p.Q3508X	nonsense	.	.	.	.	.	D	.	.	.	.	.	.	.	18.15	D	4.97	P	Novel
SCN5A	NM_198056	c.1114G>C	p.D372H	missense	.	.	D	D	D	D	H	D	D	D	D	D	0.97	27.7	D	4.73	LP	Novel
SCN10A	NM_001293306	c.1715C>T	p.P572L	missense	0.0003	0.0003	T	B	N	N	N	D	N	T	D	T	0.242	4.991	N	2.89	VUS	Reported
DSP	NM_004415	c.7046C>G	p.S2349C	missense	0.0002	0.0001	D	D	D	D	M	T	D	D	D	D	0.772	28.9	D	5.7	VUS	Reported
SCN10A	NM_001293	c.2615C	p.T872	missen	0	0	T	B	N	N	N	D	N	D	D	D	0.336	11.7	D	0.58	VU	Reporte

	306	> T	M	se														7		8	S	d
ANK2	NM_001148	c.6739C > A	p.L2247I	missense	.	.	D	.	N	D	M	T	N	D	D	D	0.314	25.2	D	5.07	VUS	Novel
DSP	NM_004415	c.6464A > G	p.D2155G	missense	0.0001	5.44E-05	D	D	D	D	M	T	D	D	D	D	0.907	28.7	D	5.37	VUS	Reported
SCN5A	NM_198056	c.4912C > T	p.R1638X	nonsense	0	0	.	.	D	D	.	.	.	.	.	.	.	41	D	4.54	P	Reported
DSP	NM_004415	c.5173C > T	p.R1725W	missense	0	0	D	D	D	D	M	T	D	T	T	D	0.497	28.3	D	5.01	VUS	Reported
RYR2	NM_001035	c.11935G > C	p.V3979L	missense	.	.	D	P	D	D	M	D	N	D	D	D	0.601	26.8	D	5.64	VUS	Novel
CACNA1C	NM_000719	c.5098G > A	p.G1700S	missense	0	0	D	D	N	D	M	D	D	D	D	D	0.777	25.9	D	4.4	VUS	Reported
SCN4A	NM_000334	c.307T > G	p.F103V	missense	0.0001	5.56E-05	D	P	D	D	M	D	D	D	D	D	0.919	25.1	D	4.23	VUS	Reported
KC	NM_0	c.24	p.Y	mis	0.00	0.0	D	B	D	D	.	D	D	T	T	D	0.5	22	D	3.	V	Rep

NE5	12282	1T > C	81H	sense	85	071											89	.7		5	US	orted
SCN4A	NM_000334	c.1666C > T	p.L556F	missense	.	.	T	B	D	D	M	D	D	D	D	D	0.652	23.3	D	3.24	VUS	Novel
KCNE3	NM_005472	c.241C > T	p.R81C	missense	0.0001	0.0002	D	B	D	D	M	D	D	D	D	D	0.474	20.9	D	3.3	VUS	Reported
HCN4	NM_005477	c.584C > T	p.A195V	missense	0.0048	0.0049	D	D	.	N	N	D	N	D	D	D	0.271	23.5	D	2.49	VUS	Reported

EAS = East Asian; ACMG = American College of Medical Genetics and Genomics

Table S5. Clinical and ECG characteristics of the included subjects classified by *SCN5A* variants.

Feature	*SCN5A* genetic positive (n = 11)	*SCN5A* genetic negative (n = 48)	P-value
Male (n, %)	10 (90.91%)	40 (83.33%)	0.869
Age at diagnosis (years)	47 (34-54)	42 (35-53)	0.893
Spontaneous Type 1 ECG (n, %)	3 (27.27%)	16 (33.33%)	0.976
Symptomatic patients (n, %)	5 (45.45%)	28 (58.33%)	0.660
Documented VT/VF (n, %)	0 (0.00%)	3 (27.27%)	1.000
Syncope (n, %)	5 (45.45%)	16 (33.33%)	0.683
ICD (n, %)	1 (9.09%)	4 (8.33%)	1.000
Heart rate (bpm)	70 (66-76)	70 (62-76)	0.619
P-wave duration (ms)	112 (92-120)	98 (87-102)	0.038*
QRS duration (ms)	102 (90-114)	100 (90-109)	0.495
T-wave duration (ms)	200 (120-220)	160 (100-199)	0.165
PR interval (ms)	162 (148-209)	161 (136-178)	0.298
QT interval (ms)	394 (380-414)	384 (360-400)	0.167
QTc interval (ms)	432 (400-447)	410 (389-421)	0.087
P-wave axis (deg)	62 (35-71)	58 (45-66)	0.938
R-wave axis (deg)	42 (6-85)	47 (23-62)	0.912
QRS axis (deg)	53 (-6-87)	46 (19-62)	0.661
T-wave axis (deg)	60 (48-99)	53 (24-66)	0.081
R-wave Amplitude in lead V1 (mV)	0.25 (0.10-0.35)	0.15 (0.08-0.30)	0.369
R-wave Amplitude in lead V5 (mV)	1.30 (1.20-1.80)	1.44 (0.99-1.75)	0.869
S-wave Amplitude in lead V1 (mV)	0.66 (0.45-1.19)	0.80 (0.41-1.08)	0.758
S-wave Amplitude in lead V5 (mV)	0.43 (0.30-0.64)	0.30 (0.11-0.50)	0.215
RV5+SV1(mV)	1.87 (1.60-3.13)	2.22 (1.52-2.69)	0.726
RV1+SV5(mV)	0.76 (0.55-0.90)	0.49 (0.31-0.72)	0.037*

For categorical variables, the data were presented as number (%). For continuous variables, the data were expressed as median (Q1–Q3). *P < 0.05

ECG = electrocardiogram; VT = ventricular tachycardia; VF = ventricular fibrillation; ICD = implantable cardioverter defibrillator; deg = degree.

Table S6. Clinical and ECG characteristics of different ECG groups classified by mutation pathogenicity.

Feature	Type 1 BrS ECG (n = 19)				Type 2/3 BrS ECG (n = 40)			
	P/LP (n = 4)	VUS (n = 3)	Negative (n = 12)	P-value	P/LP (n = 4)	VUS (n = 14)	Negative (n = 22)	P-value
Male (n, %)	4 (100%)	3 (100%)	12 (100%)	-	3 (75%)	12 (85.71%)	16 (72.73%)	0.753
Age at diagnosis (years)	49 (43-62)	47 (32-)	45 (30-61)	0.686	53 (42-64)	40 (30-46)	42 (36-55)	0.151
Symptomatic patients (n, %)	3 (75%)	2 (66.67%)	8 (66.67%)	1.000	0 (0%)	6 (42.86%)	14 (63.64%)	0.058
Documented VT/VF (n, %)	0 (0%)	0 (0%)	0 (0%)	-	0 (0%)	1 (7.14%)	2 (9.09%)	1.000
Syncope (n, %)	2 (50%)	1 (33.33%)	3 (25%)	0.781	0 (0%)	3 (21.43%)	12 (54.55%)	0.052
ICD (n, %)	0 (0%)	1 (33.33%)	1 (8.33%)	0.333	0 (0%)	2 (14.29%)	1 (4.55%)	0.673
Heart rate (bpm)	74 (69-80)	62 (53-)	71 (66-75)	0.151	75 (73-92)	70 (63-76)	63 (60-76)	0.252
P-wave duration (ms)	118 (107-128)	100 (80-)	99 (86-104)	0.085	103 (77-115)	97 (89-103)	92 (86-104)	0.897
QRS duration (ms)	120 (110-146)	120 (114-)	99 (91-112)	0.005*	97 (90-101)	95 (90-104)	100 (86-107)	0.980
T-wave duration (ms)	200 (170-3800)	200 (180-)	179 (100-195)	0.079	110 (100-180)	171 (130-205)	155 (100-196)	0.300
PR interval (ms)	194 (168-211)	160 (144-)	168 (141-187)	0.252	164 (129-180)	166 (157-181)	146 (126-174)	0.145
QT interval (ms)	383 (356-407)	394 (380-)	382 (352-396)	0.096	385 (377-455)	385 (363-417)	390 (360-416)	0.878
QTc interval (ms)	433 (403-436)	410 (400-)	415 (400-419)	0.394	429 (415-563)	390 (382-428)	408 (388-428)	0.086
P-wave axis (deg)	49 (33-62)	48 (48-)	57 (48-71)	0.538	59 (41-78)	63 (50-72)	60 (25-66)	0.495
R-wave axis (deg)	15 (-32-50)	48 (45-)	59 (42-64)	0.022*	50 (-10-98)	37 (14-62)	46 (19-60)	0.794
QRS axis (deg)	24 (-32-123)	76 (0-)	61 (47-79)	0.534	35 (-19-98)	32 (14-62)	43 (19-55)	0.879
T-wave axis (deg)	17 (-5-65)	48 (21-)	56 (33-66)	0.306	54 (23-89)	59 (37-81)	54 (19-65)	0.371
R-wave Amplitude in lead V1 (mV)	0.15 (0.05-0.29)	0.05 (0.00-)	0.29 (0.04-0.48)	0.657	0.28 (0.16-0.69)	0.12 (0.10-0.24)	0.13 (0.09-0.30)	0.199

R-wave Amplitude in lead V5 (mV)	1.12 (1.02-1.41)	1.29 (0.80-)	1.43 (1.09-1.85)	0.370	1.39 (0.63-1.72)	1.37 (1.05-1.85)	1.47 (0.87-1.92)	0.754
S-wave Amplitude in lead V1 (mV)	0.24 (0.03-0.64)	0.45 (0.10-)	1.00 (0.59-1.21)	0.010*	0.73 (0.56-1.68)	0.81 (0.62-1.12)	0.63 (0.28-1.09)	0.449
S-wave Amplitude in lead V5 (mV)	0.68 (0.26-1.17)	0.64 (0.45-)	0.20 (0.06-0.47)	0.020*	0.37 (0.15-0.45)	0.30 (0.14-0.50)	0.29 (0.14-0.46)	0.940
RV5+SV1(mV)	1.49 (1.19-1.78)	1.74 (0.90-)	2.53 (1.87-2.90)	0.042*	2.11 (1.20-3.39)	2.23 (1.71-2.99)	2.14 (1.44-2.78)	0.880
RV1+SV5(mV)	0.83 (0.36-1.40)	0.69 (0.45-)	0.50 (0.34-0.72)	0.203	0.72 (0.50-0.87)	0.50 (0.29-0.78)	0.49 (0.37-0.80)	0.462

For categorical variables, the data were presented as number (%). For continuous variables, the data were expressed as median (Q1–Q3).

BrS = brugada syndrome; ECG = electrocardiogram; VT = ventricular tachycardia; VF = ventricular fibrillation; ICD = implantable cardioverter defibrillator; deg = degree.

P/LP = patients with pathogenic or likely pathogenic mutations; VUS = patients with uncertain significance variants; Negative = patients without variants.
